# Supplementary material for: Quantification of ongoing APOBEC3A activity in tumor cells by monitoring RNA editing at hotspots
Source: Nat Commun. 2020 Jun 12;11:2971. doi: 10.1038/s41467-020-16802-8 (PMC7293259; doi:10.1038/s41467-020-16802-8)
Supplement: Supplementary file 1 — Supplementary Information [file 41467_2020_16802_MOESM1_ESM.pdf]

Supplementary information for

# Quantification of ongoing APOBEC3A activity in tumor cells by monitoring RNA editing at hotspots

Pégah Jalili, Danae Bowen, Adam Langenbucher, Shinho Park, Kevin Aguirre, Ryan B. Corcoran, Angela G. Fleischman, Michael S. Lawrence, Lee Zou & Rémi Buisson

## SUPPLEMENTARY METHODS

### Computational APOBEC analyses

Paired tumor-normal sequencing data from The Cancer Genome Atlas (TCGA) was analyzed to study molecular correlates of APOBEC enzyme activity at the DNA and RNA levels. Data from whole-exome DNA sequencing (WXS), whole-genome DNA sequencing (WGS), and RNA sequencing (RNA-Seq), was included in the analysis, all aligned to human genome build hg19. The TCGA WGS and WXS dataset was as described previously [1]. Mutation calls from whole-exome sequencing (WXS) were obtained from the TCGA Unified Ensemble "MC3" Call Set [2], the public, open-access dataset of somatic mutation calls produced by the MC3 calling effort ("Multi-Center Mutation Calling in Multiple Cancers"), downloaded from the following link: http://www.synapse.org/#!Synapse:syn7214402/wiki/405297 (The results here are in whole or part based upon data generated by the TCGA Research Network: http://cancergenome.nih.gov/ as outlined in the TCGA publications guidelines http://cancergenome.nih.gov/publications/publicationguidelines) Following the filtering procedure that was used for the PanCanAtlas project, the MC3 dataset was filtered to include only "PASS" variants, which removes patients that were subjected to whole-genome amplification (WGA), as well as the acute myeloid leukemia (LAML) cohort. This yielded a final cohort of 9023 patients covering 32 tumor types. Mutation calls from whole-genome sequencing (WGS) were from TCGA and additional published WGS datasets [3,4], all restricted to somatic single-nucleotide variants (SSNVs) and excluding patients with fewer than 500 SSNVs in the genome, yielding a final WGS dataset comprising 1686 unique patients spanning 27 tumor types, listed in Supplementary Table 1.

Two separate analyses were carried out:

Analysis 1: Comparison of APOBEC DNA mutation signatures and RNA expression levels

Analysis 2: Identification of APOBEC RNA editing hotspots

Each analysis used a different subset of the TCGA data. Analysis 1 compared somatic mutation calls from TCGA DNA whole-genome sequencing (WGS) and APOBEC expression level estimates from TCGA RNA sequencing (RNA-Seq). Analysis 2 compared DNA and RNA raw sequencing data at the single-base level, comparing across trios of normal DNA, tumor DNA, and tumor RNA from the same patient, using TCGA DNA whole-exome sequencing (WXS) and RNA-Seq.

**Comparison of APOBEC DNA mutation signatures and RNA expression levels**

Mutation signatures were decomposed by Non-negative Matrix Factorization (NMF, k=8) as described previously [1], revealing a set of signatures corresponding to known mutational processes, including APOBEC. For each patient, we calculated the fractional contribution of the APOBEC mutation signature (reported as "frac_apobec" in Supplementary Table 1 and shown as a percentage on the y-axes of Figures. 1A, 1B, 3D, and Supplementary Figures 1, and 8A.) Next, to distinguish the contributions of APOBEC3A (A3A) and APOBEC3B (A3B), we employed an established metric based on the observation that A3A prefers a pyrimidine at the "minus-2" position, whereas A3B prefers a purine there [5]. Accordingly we calculated the A3A and A3B character of each patient's mutations as the fraction of cytosine mutations occurring in the contexts YTC or RTC, respectively (reported as "YTC_C" and "RTC_C" in Supplementary Table 1), as described previously [1]. This measurement of A3A character is shown on the y-axes of Figures 1C and 1D. To obtain a directional metric of each patient's overall A3A or A3B mutation character, we calculated YTC_C minus twice RTC_C. This metric (reported as "ABchar" in Supplementary Table 1 and shown on the x-axes of Figures 1A, 1B, 3D, and Supplementary 1, and 3A) is negative (left-hand side of plot) for samples dominated by A3B mutagenesis, which preferentially mutates Cs in the context RTC, and positive (right-hand side of plot) for samples dominated by A3A mutagenesis, which preferentially mutates Cs in the

context YTC. Figure. 1A-B shows a subset of the full 1686-patient WGS dataset, with bladder, breast, cervical, endometrial, head-and-neck, and lung cancers included due to the high prevalence of APOBEC mutations in these diseases, and gliomas (glioblastoma multiforme and low-grade gliomas) and chronic lympocytic leukemia (CLL) included as representative APOBEC-negative tumor types. The entire 1686-patient WGS dataset is shown in Supplementary Figure 1 and Supplementary Table 1. In Supplementary Figure 1, tumor types are organized into rough qualitative categories of "APOBEC3A" (e.g. bladder, cervical, lung, in which many samples have high levels of APOBEC mutation, always with predominant A3A character), "APOBEC3A/B" (breast cancer, in which also many samples have high levels of APOBEC mutation, but some samples show predominant A3A character and others A3B), "APOBEC3B" (sarcoma, kidney, in which many samples have high levels of APOBEC character, but only A3B-dominated samples are seen), and "APOBEC3-negative" (e.g. glioma, CLL, in which most samples have <10% APOBEC mutations). To quantify the prevalence of DNA hairpin mutations, in each sample, the fraction of TpC mutations occurring at DNA stem-loop sites was calculated as described previously [1] and is shown as a red colorscale in Figures 1B, 1D, and 3E (reported as "frac_hairpin" in Supplementary Table 1). For TCGA samples with RNA-Seq data available, RNA expression levels of A3A and A3B were quantified by detection of transcribed sequences that uniquely distinguish A3A or A3B from each other and from other APOBECs, as described previously [1]. These counts are reported in Supplementary Table 1 as "RNA_APOBEC3A_unq" and "RNA_APOBEC3B_unq" and are shown on the x-axes of Figures 1C, 1D, and 3E (A3A) and Supplementary Figure 8B (A3B).

### Identification of APOBEC RNA editing hotspots

Having surveyed the landscape of APOBEC mutation signatures and expression levels, we next aimed to identify A3A-associated RNA editing hotspots that could be employed for detection of ongoing A3A activity in cells. A3A was previously reported to edit RNA hairpins that have the

tetraloop sequence CAUC, with editing at the underlined C [6]. To confirm this observation and possibly find additional edited sites, we scanned the entire transcriptome for RNA editing hotspots. Our approach was designed to be extremely conservative, and used a custom multi-step filtering approach to remove variable positions resulting from DNA polymorphisms, DNA mutations, non-APOBEC-related RNA editing, and common sequencing noise and alignment artifacts. We focused on bladder, cervical, and head-and-neck cancer as highly A3A-enriched tumor types. Within this cohort, we selected a set of 50 patients with matched DNA (WXS) and RNA-Seq data available. The 50 patients (listed in Supplementary Table 2) were selected to include 25 patients with high levels and 25 with low levels of A3A expression, referred to as "APO-High" and "APO-Low", respectively. For each patient, we downloaded a trio of BAMs from the GDC Data Portal (https://portal.gdc.cancer.gov/): the DNA WXS BAMs from the paired normal and tumor samples, and the RNA-Seq BAM from the tumor. As a control "APO-Neg" cohort, we chose gastric cancer as a representative largely APOBEC-negative disease and selected 27 patients with undetectable A3A mRNA, also listed in Supplementary Table 2. As our universe of possible editing sites, we considered the ~8 million TpC sites contained with GENCODE-annotated exonic regions. For positions in which the TpC is located on the anti-genomic strand (i.e. a GpA on the genomic strand), we reverse-complemented the data, so as to consider C as the reference base for all positions. We retrieved base counts (#A, #C, #G, #T) for each site from the normal-DNA, tumor-DNA and tumor-RNA BAM of each patient, excluding duplicate or mapping-quality-zero reads, and excluding base calls with base quality <10. This yielded a matrix of base counts at each position in each BAM, which was then filtered by a series of steps as follows. First, the published "Panel of Normals" (PoN) approach was used to mask positions of common germline variation or sequencing noise, as described previously [2]. In short, a panel of 8000 TCGA normal samples were aggregated, and the total fraction of non-reference (i.e. non-C) base calls at each position was calculated, summing across the PoN. Positions at which >0.2% of base calls in the PoN were non-C were

considered "variable" and masked from analysis. Next, to exclude positions subject to non-A3A-related RNA editing, we aggregated RNA-Seq data across the 27 A3A-negative gastric cancer patients, measured the total number of reads at each position, and counted the total "edited" fraction of these reads (i.e. those carrying a T base call). Any site with >0.5% editing (or covered by <4000 total reads) in the A3A-negative gastric cohort was masked from analysis. Next, we masked additional sites in each patient of the bladder/cervical/HN cohort. To exclude sites with insufficient sequencing coverage, sites with <14 reads in any of that patient's three BAMs were masked from analysis. To exclude sites with a patient-specific DNA mutation or polymorphism, sites with >1 non-reference base call (A/G/T) in either the normal or tumor DNA sample were masked from analysis. To exclude sites that are noisy in the RNA-Seq, we counted the number of purine (A or G) base calls (i.e. neither reference C nor edited T), and sites with >1 purine base call (representing at least 0.2% of total calls) were masked from analysis. After this process of masking sites in individual patients, we fully masked "repeat offender" sites (those masked in 10 or more patients), masking them in *all* patients. We then finally examined RNA C->U editing in the bladder/cervical/HN cohort, calculating the mean editing fraction (ignoring masked sites) in the APO-High and APO-Low cohorts. Supplementary Figure 3 shows a scatter plot of all candidate sites, with the top 50 sites highlighted with red points, and gene names shown for the top 20 sites. We selected the top 50 sites (listed in Supplementary Table 3) as likely hotspots of RNA editing by A3A. Out of these top sites, 48/50 had the TpC on the transcribed strand, and the other 2/50 had the TpC on the nontranscribed strand. Sites were classified by hairpin characteristics such as stem strength, and loop length and UpC motif positioning as described previously [1] but using spliced transcript sequences (GENCODE) instead of unspliced DNA sequences. The top 50 edited sites tended to be in strongly paired hairpins (Supplementary Figure 7). This enrichment was extremely clear when comparing to the background distribution of hairpin strength among the ~8M TpC sites in the transcriptome ($p = 1.7 \times 10^{-21}$, t-test). Manual inspection of sequencing reads supporting the top

RNA editing hotspots (Supplementary Figures 4, 5, and 6) confirmed that these editing events are occurring in the RNA of APO+ samples, with no trace of the C->T mutation visible in the DNA, or in the RNA of APO- samples. The top two RNA editing sites, in the genes DDOST and CYFIP1, were chosen for further validation *in vitro* and in clinical samples.

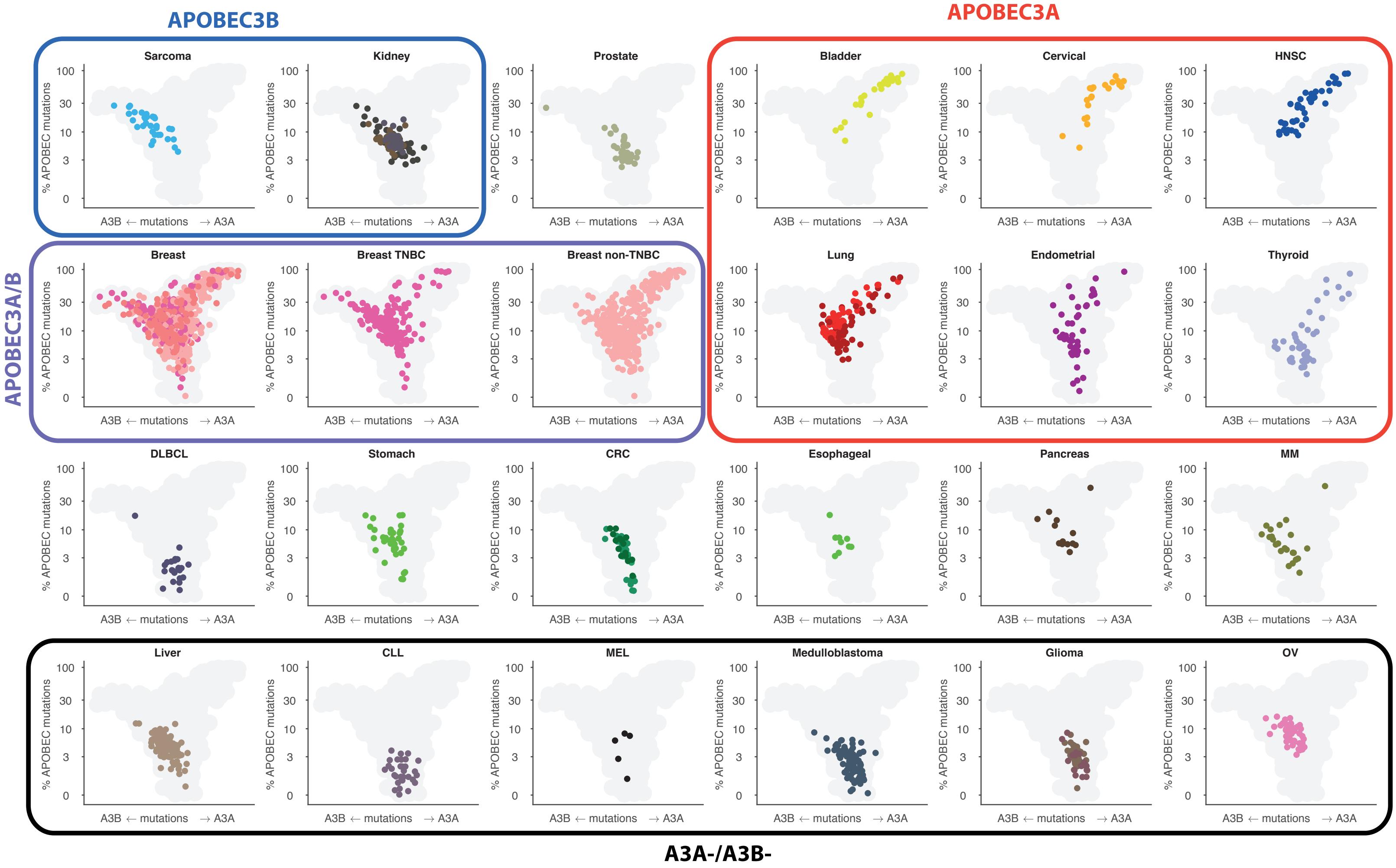


**Supplementary Figure 1:** A cohort of patient tumor samples sequenced by WGS (in TCGA and other projects) were analyzed for their mutation frequency in the TpC motif. Patients were stratified by cancer types and plotted by their level of mutations in the TpC motif ("APOBEC-signature mutations") and their mutation frequency in RTC versus YTC sequences (indicative of A3B and A3A character, respectively).

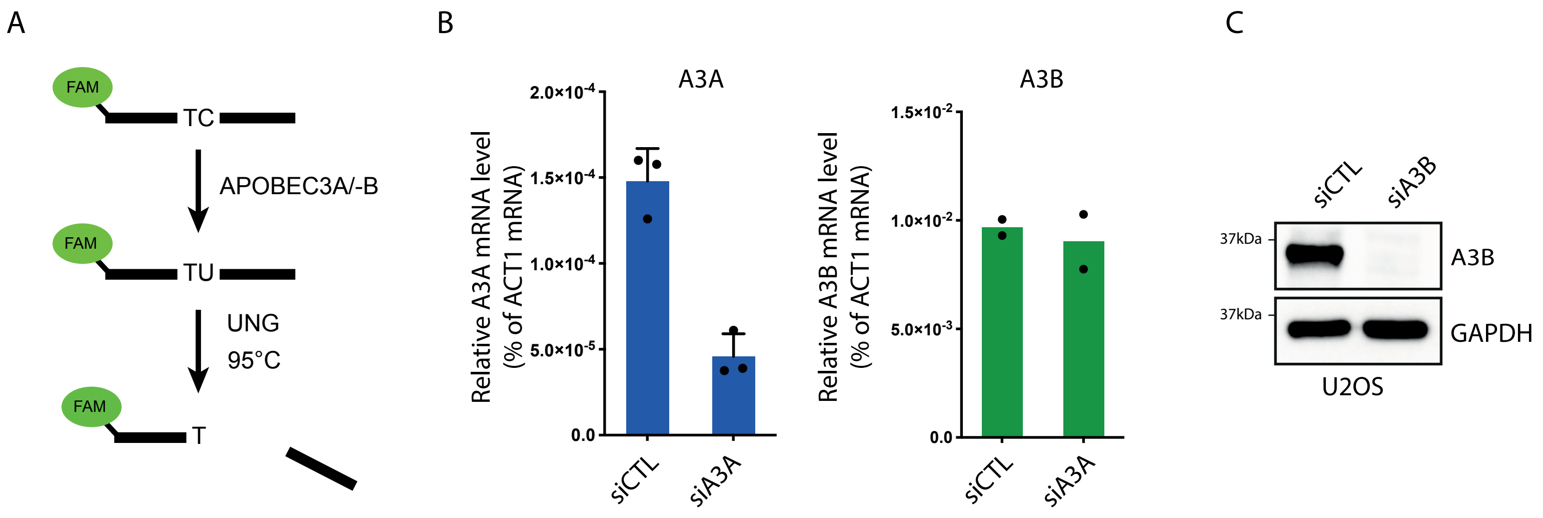


**Supplementary Figure 2: A.** Schematic diagram of the DNA deaminase activity assay for A3A and A3B. **B.** A3A and A3B mRNA levels in BICR6 cells were monitored by RT-qPCR after A3A siRNA knockdown. Error bar: S.D. (n = 3). **C.** A3B protein level was determined by Western blotting in BICR6 cells.

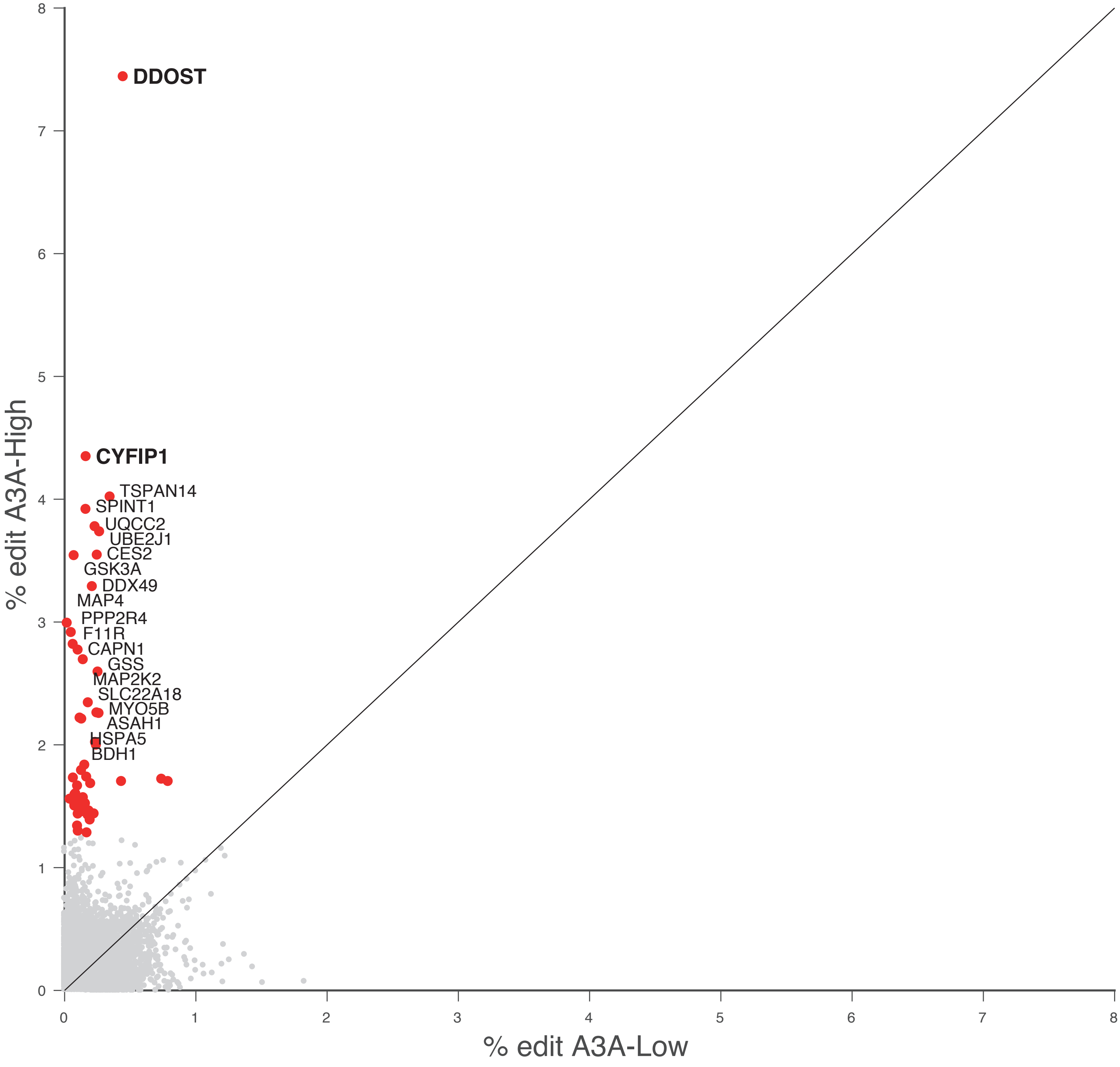


**Supplementary Figure 3:** Top RNA editing hotspots in a cohort of 50 bladder, cervical, and head-and-neck tumors from TCGA. All TpC sites in the transcriptome (GENCODE hg19) were surveyed for C->U RNA editing activity, after filtering to mask polymorphic, noisy, or insufficiently covered sites (see Methods). Remaining sites were ranked by the average percent C->U editing in the 25 A3A-high members of the cohort (y-axis). The top 50 editing hotspots are highlighted with red points, and the top 20 of these are shown with the names of the genes that the hotspots occur in. Editing levels in the 25 A3A-Low members of the cohort (x-axis) was low by comparison.

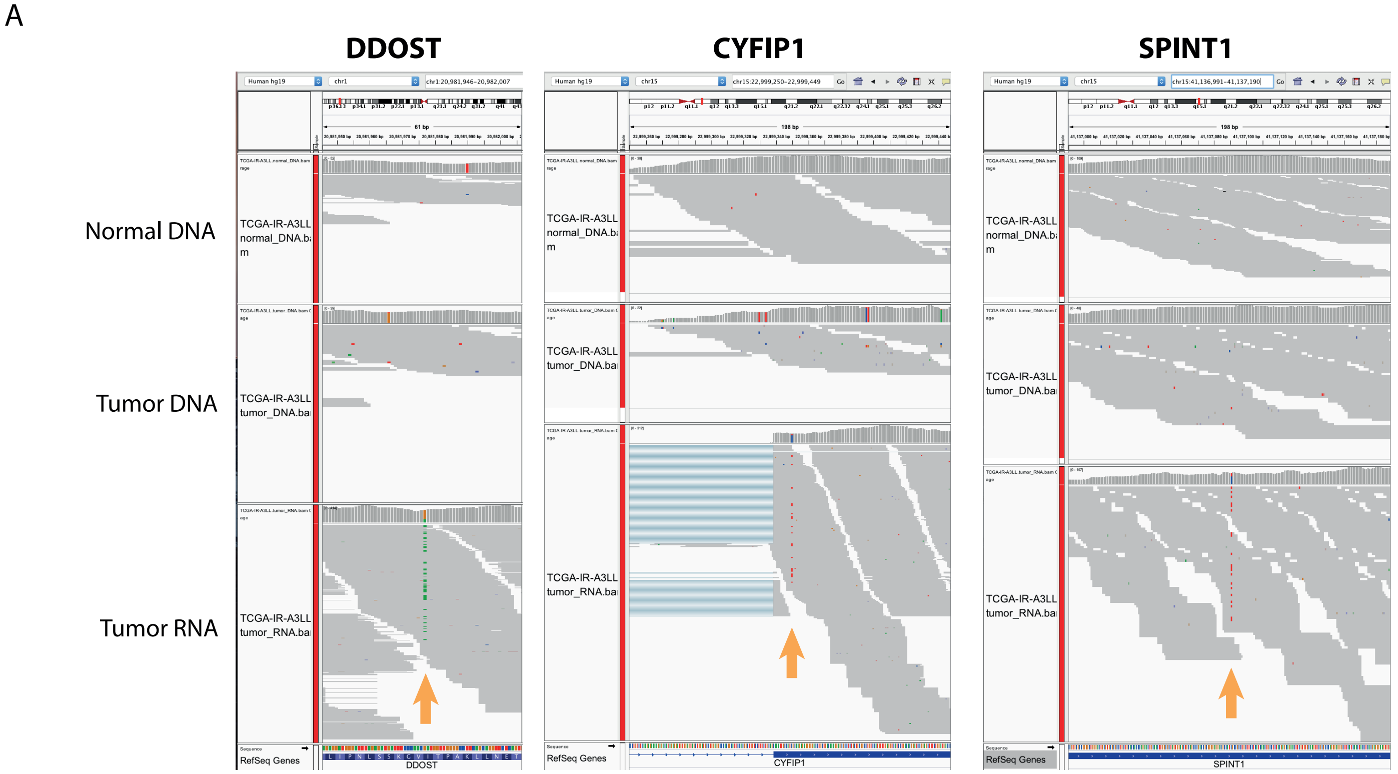


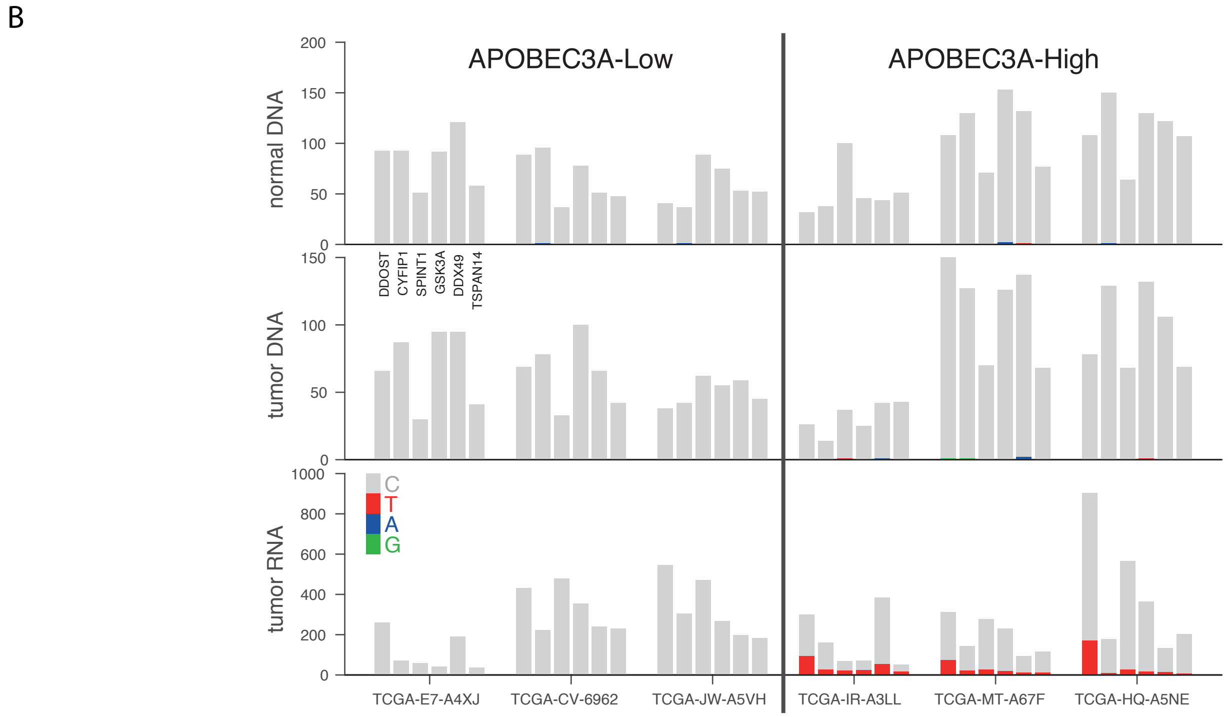


**Supplementary Figure 4: A.** Examples of sequencing data supporting RNA editing hotspots. Three of the top C->U RNA editing hotspots, in the genes DDOST, CYFIP1, and SPINT1, are shown in a TCGA patient with very strong editing levels (cervical patient TCGA-IR-A3LL). Aligned sequencing reads (sorted by start position) are shown for the normal DNA (top), tumor DNA (middle), and tumor RNA (bottom) from this patient. The positions of the RNA editing events are shown with orange arrows. Sequencing reads in the DNA are completely grey (reference sequence), whereas a proportion of reads in the RNA are colored (green=A for DDOST, with its TpC site on the antigenomic strand; and red=T for CYFIP1 and SPINT1, with their TpC sites on the genomic strand), constituting clear evidence of RNA editing. **B.** Read counts supporting RNA editing hotspots. Six of the top C->U RNA editing hotspots, in the genes DDOST, CYFIP1, SPINT1, GSK3A, DDX49, and TSPAN14, are shown, in six TCGA patients from the bladder, cervical, and head-and-neck cohort: three patients with low A3A levels (left three groups of bars), and three patients with high A3A levels (right three groups of bars). Bars show raw readcounts from each patient's normal DNA, tumor DNA, and tumor RNA bams. C is seen nearly exclusively in all patients' DNA, and in the RNA of the A3A-Low patients, whereas a proportion of T is seen in the RNA of the A3A-High patients, ranging from low (~1%) to high (~30%) levels.

# DDOST

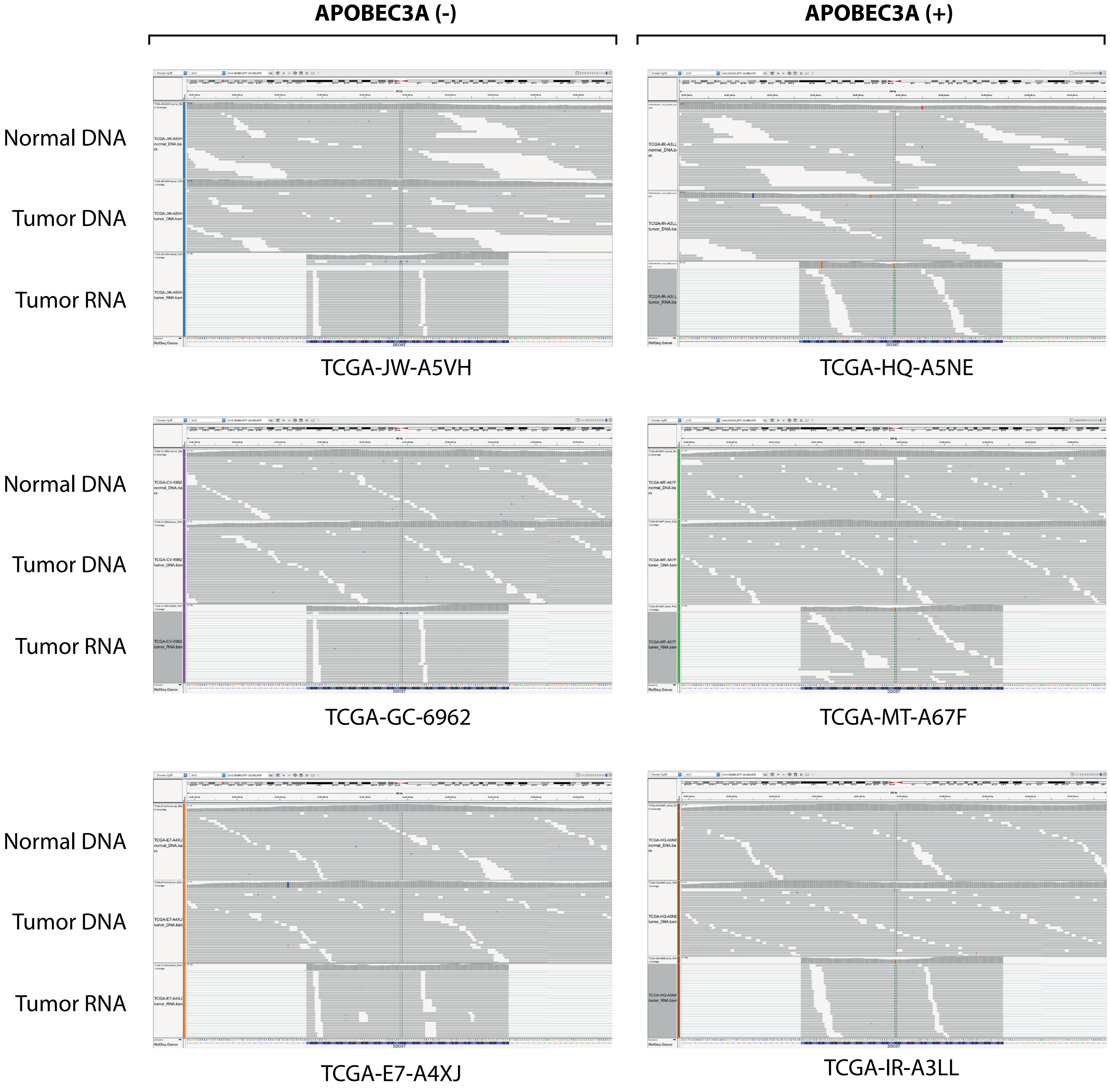


**Supplementary Figure 5:** IGV screenshots supporting DDOST RNA editing hotspots. Six TCGA patients from Supplementary Fig. 4B are shown (three A3A-Low (APOBEC3A(-)); three A3A-High (APOBEC3A(+)). In each IGV screenshot, the idicated patient's three BAMs are shown: normal DNA (top), tumor DNA (middle), and tumor RNA (bottom). Aligned sequencing reads (sorted by base) are shown. The position of the RNA editing hotspot is flanked by dashed vertical lines. In each case, strong evidence of RNA editing is seen in the A3A-High RNA data (APOBEC3A (+)), but not in the A3A-Low RNA data ((APOBEC3A (-)), and not in any of the DNA data.

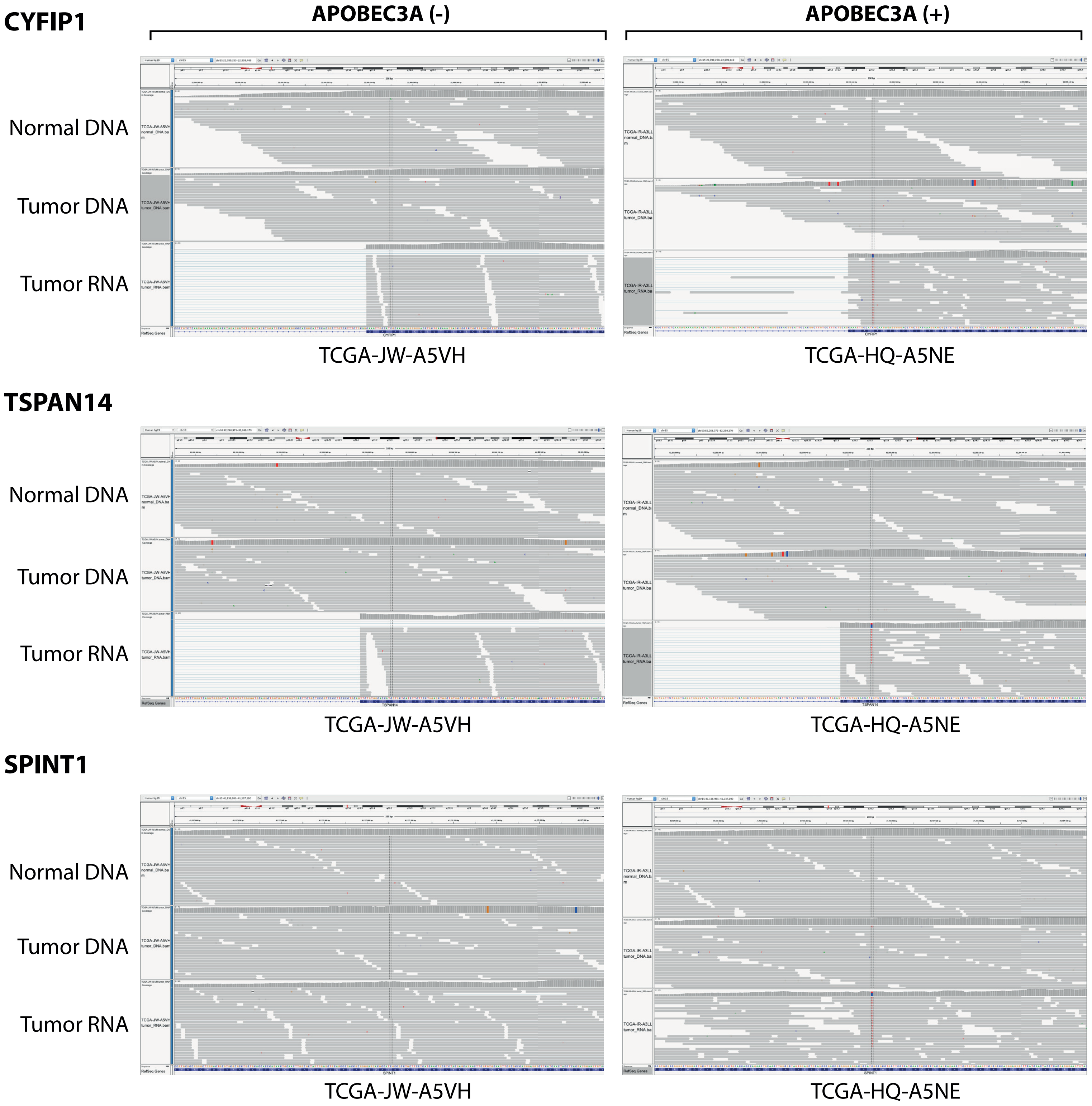


**Supplementary Figure 6:** IGV screenshots supporting of 3 other top RNA editing hotspots. For each hotspot, two TCGA patients from Supplementary Fig. 4B are shown (one A3A-Low column (APOBEC3A(-)); one A3A-High column (APOBEC3A(+)). In each IGV screenshot, the patient's three BAMs are shown: normal DNA (top), tumor DNA (middle), and tumor RNA (bottom). Aligned sequencing reads (sorted by base) are shown. The position of the RNA editing hotspot is flanked by dashed vertical lines. In each case, strong evidence of RNA editing is seen in the A3A-High RNA data (APOBEC3A (+)), but not in the A3A-Low RNA data ((APOBEC3A (-)), and not in any of the DNA data.

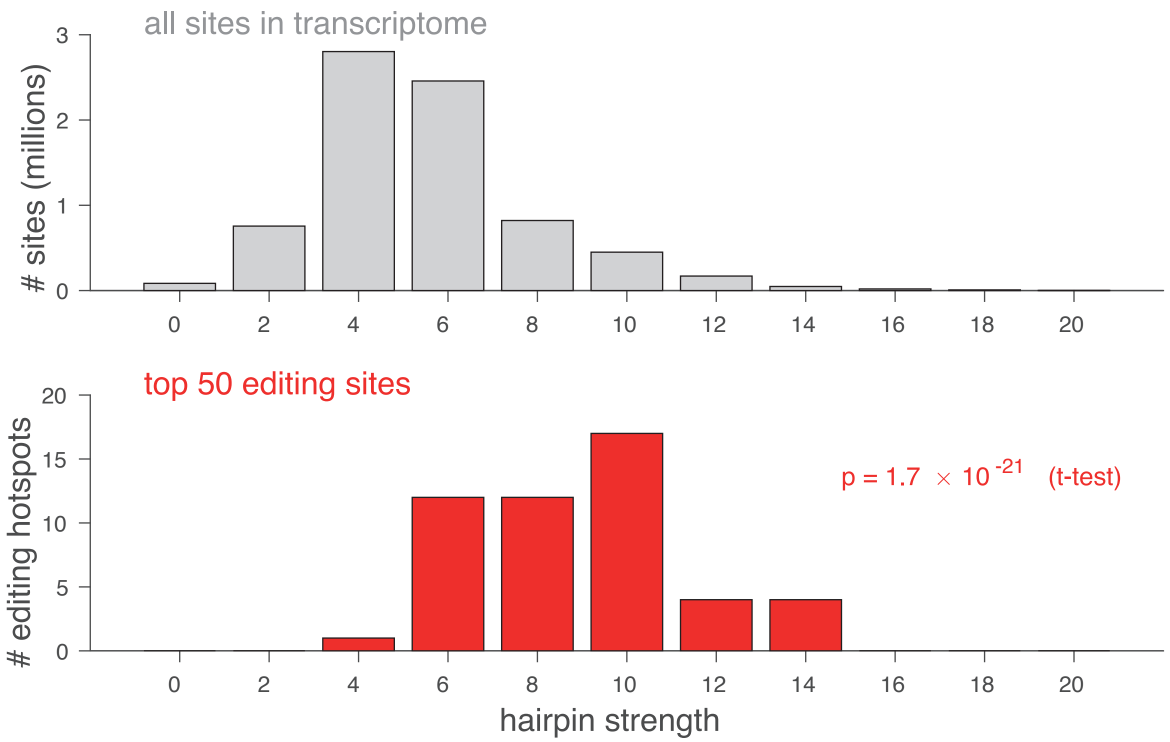


**Supplementary Figure 7:** Top RNA editing hotspots tend to be in hairpins. All TpC sites in the transcriptome (GENCODE hg19) were classified by their potential hairpin strength, measuring the pairing ability of the flanking RNA sequences (see Methods). The top panel (grey bars) shows the distribution of hairpin strength for all ~8M TpC sites in the transcriptome. The lower panel (red bars) shows the distribution of hairpin strength for the top 50 C->U RNA editing hotspots identified in the analysis of the bladder, cervical, and head-and-neck tumor from TCGA. The RNA editing hotspots show a highly significant enrichment in strong hairpins (p = 1.7 x 10-21, unpaired two-sample Student's t-test).

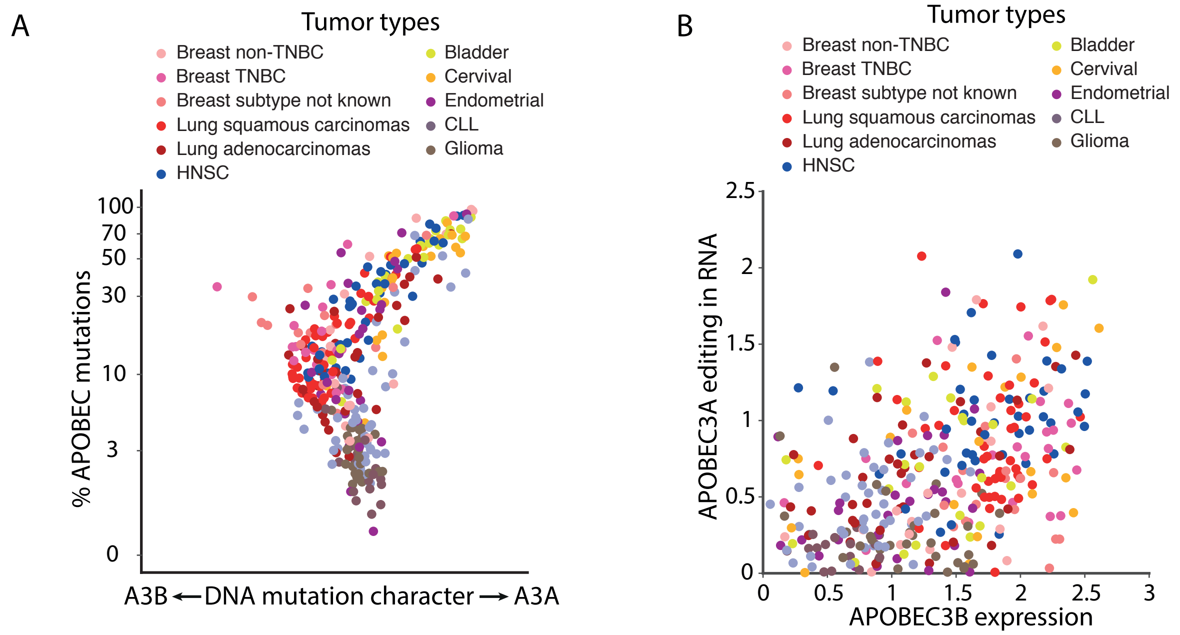


**Supplementary Figure 8: A.** Indicated patient tumor types were superimposed on the patients from Figure 1B for whom RNA-Sequencing data was available. **B.** Patients' tumor samples were plotted according to their A3B expression level and RNA editing level. Color-codes indicate patients' tumor types.

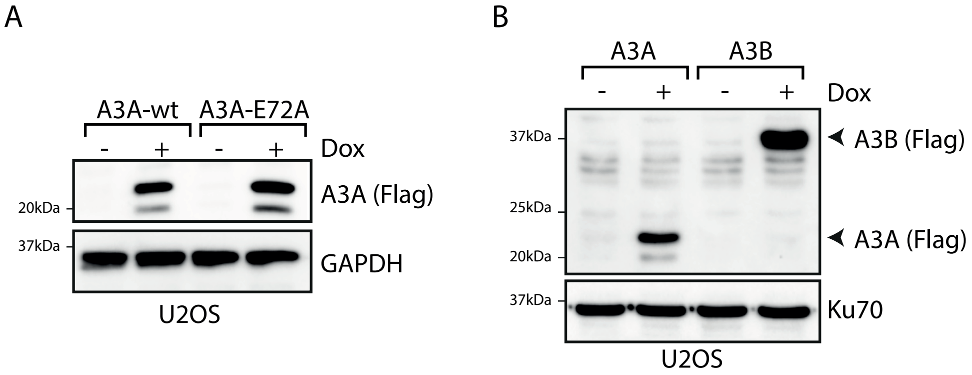


**Supplementary Figure 9: A-B.** A3A, A3A-E72A, and A3B levels in U2OS-derived cells were monitored by Western blot after 48h of DOX treatment.

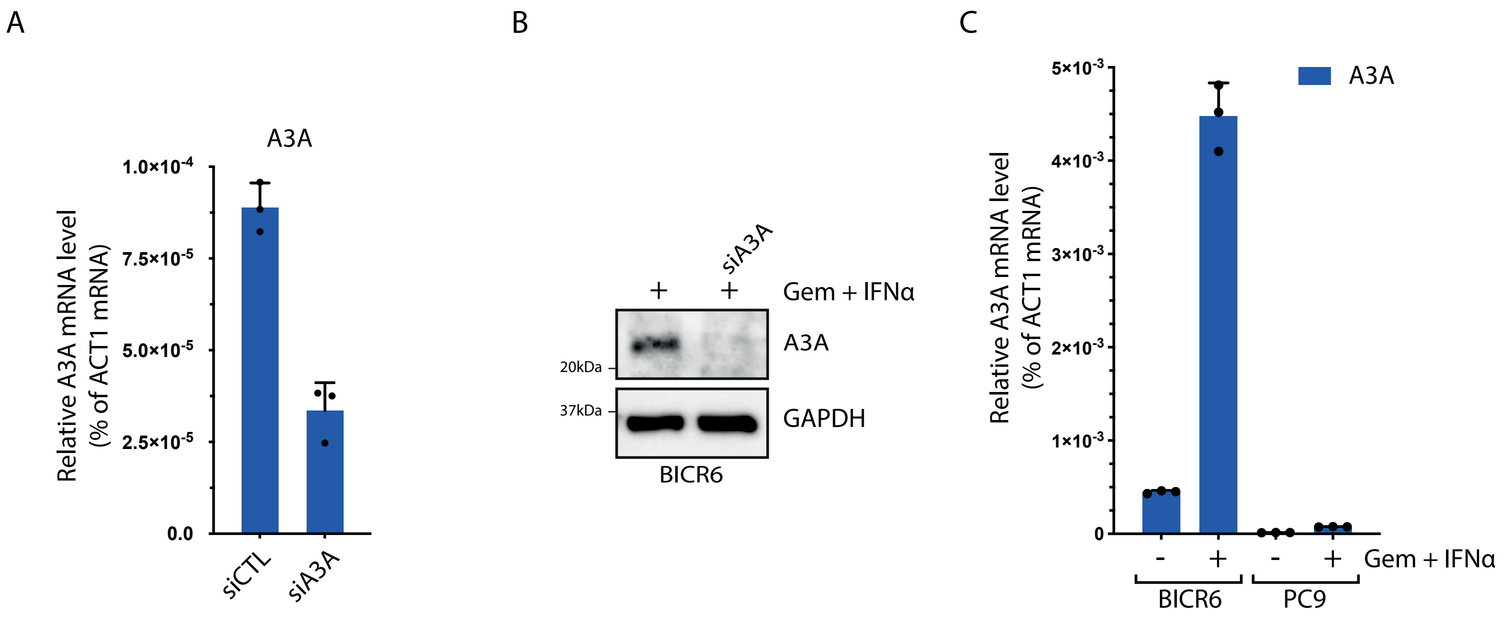


**Supplementary Figure 10: A.** A3A mRNA levels in BICR6 cells were monitored by RT-qPCR after A3A siRNA knockdown. Error bar: S.D. (n = 3). **B**. Western Blot analysis of A3A in BICR6 cells after A3A knowndown and 48h treatment with Gemcitabine (0.5 µM) and Interferon-αA/D (750U.ml$^{-1}$). **C.** A3A mRNA levels in BICR6 and PC9 cells treated for 48h with Gemcitabine and Interferon-αA/D or left untreated. Error bar: S.D. (n = 3).

## SUPPLEMENTARY REFERENCES


1. Buisson, R. *et al.* Passenger hotspot mutations in cancer driven by APOBEC3A and mesoscale genomic features. *Science (80-. ).* **364**, eaaw2872 (2019).
2. Ellrott, K. *et al.* Scalable Open Science Approach for Mutation Calling of Tumor Exomes Using Multiple Genomic Pipelines. *Cell Syst.* **6**, 271-281.e7 (2018).
3. Alexandrov, L. B. *et al.* Signatures of mutational processes in human cancer. *Nature* **500**, 415–421 (2013).
4. Nik-Zainal, S. *et al.* Landscape of somatic mutations in 560 breast cancer whole-genome sequences. *Nature* **534**, 47–54 (2016).
5. Chan, K. *et al.* An APOBEC3A hypermutation signature is distinguishable from the signature of background mutagenesis by APOBEC3B in human cancers. *Nat. Genet.* **47**, 1067–72 (2015).
6. Sharma, S. *et al.* APOBEC3A cytidine deaminase induces RNA editing in monocytes and macrophages. *Nat. Commun.* **6**, 6881 (2015).
