# Supplementary material for: Quantification of ongoing APOBEC3A activity in tumor cells by monitoring RNA editing at hotspots
Source: Nat Commun. 2020 Jun 12;11:2971. doi: 10.1038/s41467-020-16802-8 (PMC7293259; doi:10.1038/s41467-020-16802-8)
Supplement: Supplementary file 3 — Reporting Summary [file 41467_2020_16802_MOESM3_ESM.pdf]

nature research

Corresponding author(s): Rémi Buisson
Lee Zou
Michael Lawrence

Last updated by author(s): May 5, 2020

Reporting Summary

Nature Research wishes to improve the reproducibility of the work that we publish. This form provides structure for consistency and transparency in reporting. For further information on Nature Research policies, see Authors & Referees and the Editorial Policy Checklist.

Statistics

For all statistical analyses, confirm that the following items are present in the figure legend, table legend, main text, or Methods section.

n/a	Confirmed	
☐	☒	The exact sample size (n) for each experimental group/condition, given as a discrete number and unit of measurement
☐	☒	A statement on whether measurements were taken from distinct samples or whether the same sample was measured repeatedly
☐	☒	The statistical test(s) used AND whether they are one- or two-sided<br>*Only common tests should be described solely by name; describe more complex techniques in the Methods section.*
☒	☐	A description of all covariates tested
☒	☐	A description of any assumptions or corrections, such as tests of normality and adjustment for multiple comparisons
☐	☒	A full description of the statistical parameters including central tendency (e.g. means) or other basic estimates (e.g. regression coefficient) AND variation (e.g. standard deviation) or associated estimates of uncertainty (e.g. confidence intervals)
☐	☒	For null hypothesis testing, the test statistic (e.g. F, t, r) with confidence intervals, effect sizes, degrees of freedom and P value noted<br>*Give P values as exact values whenever suitable.*
☒	☐	For Bayesian analysis, information on the choice of priors and Markov chain Monte Carlo settings
☒	☐	For hierarchical and complex designs, identification of the appropriate level for tests and full reporting of outcomes
☒	☐	Estimates of effect sizes (e.g. Cohen's d, Pearson's r), indicating how they were calculated

Our web collection on statistics for biologists contains articles on many of the points above.

Software and code

Policy information about availability of computer code

Data collection	Open source and custom code were used to generate figure 1 and 2. Raw data used in these figures are already publicly available.<br>Gel images were acquired using a Chemidoc MP (BioRad).Tumor sequencing data analyzed in this paper is publicly available from TCGA and can be downloaded from the Genomic Data Commons website (https://portal.gdc.cancer.gov/), accession number phs000178.v11.p8
Data analysis	Gel images were quantified with ImageLab (Biorad).<br>Graphs were generated using Prim 6 (GraphPad).<br>ddPCR results were analyzed using QuantaSoft (Biorad)

For manuscripts utilizing custom algorithms or software that are central to the research but not yet described in published literature, software must be made available to editors/reviewers. We strongly encourage code deposition in a community repository (e.g. GitHub). See the Nature Research guidelines for submitting code & software for further information.

Data

Policy information about availability of data

All manuscripts must include a data availability statement. This statement should provide the following information, where applicable:

- Accession codes, unique identifiers, or web links for publicly available datasets
- A list of figures that have associated raw data
- A description of any restrictions on data availability

Tumor sequencing data analyzed in this paper is publicly available from TCGA and can be downloaded from the Genomic Data Commons website (https://portal.gdc.cancer.gov/), accession number phs000178.v11.p8. The source data underlying Figures 2a-b, 2d-e, 4c-g, 5, 6, 7 and Supplementary Figures 2b-c, 4b, 7, 9 and 10 are provided as a Source Data file.

Field-specific reporting

Please select the one below that is the best fit for your research. If you are not sure, read the appropriate sections before making your selection.

☒ Life sciences ☐ Behavioural & social sciences ☐ Ecological, evolutionary & environmental sciences

For a reference copy of the document with all sections, see nature.com/documents/nr-reporting-summary-flat.pdf

Life sciences study design

All studies must disclose on these points even when the disclosure is negative.

Sample size	No statistical methods were used for samples size. Sample sizes of at least 3 different experiments (RNA purifications) were used throughout the study.
Data exclusions	Results from qPCR with a high Ct (more than 22) for the reference gene (actin) were excluded.
Replication	All experiments have been repeated in multiple independent experiments. All key experiments have been repeated in different cell lines or using different PCR probes.
Randomization	N/A
Blinding	All key experiments have been repeated by a second person.

Reporting for specific materials, systems and methods

We require information from authors about some types of materials, experimental systems and methods used in many studies. Here, indicate whether each material, system or method listed is relevant to your study. If you are not sure if a list item applies to your research, read the appropriate section before selecting a response.

Materials & experimental systems

n/a	Involved in the study	
☐	☒	Antibodies
☐	☒	Eukaryotic cell lines
☒	☐	Palaeontology
☒	☐	Animals and other organisms
☐	☒	Human research participants
☒	☐	Clinical data

Methods

n/a	Involved in the study	
☒	☐	ChIP-seq
☒	☐	Flow cytometry
☒	☐	MRI-based neuroimaging

Antibodies

Antibodies used	The antibodies used in this study were: Flag-M2 monoclonal antibody (Sigma-Aldrich #F1804) GAPDH polyclonal antibody (EMD Millipore #ABS16), Flag polyclonal antibody (Sigma-Aldrich #F7425), APOBEC3B monoclonal antibody (Abcam #ab184990), APOBEC3A/B polyclonal antibody (Santa Cruz #sc-86289), and Ku70 monoclonal antibody (Gene Tex #GTX70271).
Validation	Antibodies specificity were confirmed by knockdown the targeted proteins with siRNA and/or by overexpressing the targeted proteins.

Eukaryotic cell lines

Policy information about cell lines

Cell line source(s)	BICR6, U2OS, RPE1-hTERT, TOV21G, PC9, OV17R, OVCAR5, SKBR3, NCI-H2347,and HEK-293T were purchased from either ATCC or Sigma-Aldrich.
Authentication	All cell lines were obtained from commercial repositories (ATTC, DSMZ, ECACC, or JHSF/JCRB). Upon receipt, the cell lines were expanded and frozen stocks were created. Stocks were further authenticated as follows: To identify cross-contaminated or synonymous lines, a panel of SNPs was profiled for each cell line (Sequenom) and a pair-wise comparison score was calculated. In addition, we performed short tandem repeat (STR) analysis (AmpFLSTR Identifiler, Applied Biosystems) and matched this to an existing STR profile generated by the providing repository. From authenticated frozen stocks, cells were not continuously kept in culture for more than 3 months. For the experiments described in this article, cell lines were not continuously kept in culture for more than 3 months.

Mycoplasma contamination	All cell lines were tested repetitively for Mycoplasma contamination
Commonly misidentified lines (See ICLAC register)	N/A

Human research participants

Policy information about studies involving human research participants

Population characteristics	patients with hematologic malignancies
Recruitment	Peripheral blood or bone marrow was obtained from patients with hematologic malignancies. All participants gave their informed consent for the studies conducted in accordance with the Declaration of Helsinki.
Ethics oversight	University of California, Irvine

Note that full information on the approval of the study protocol must also be provided in the manuscript.
