# Supplementary material for: Quantification of ongoing APOBEC3A activity in tumor cells by monitoring RNA editing at hotspots
Source: Nat Commun. 2020 Jun 12;11:2971. doi: 10.1038/s41467-020-16802-8 (PMC7293259; doi:10.1038/s41467-020-16802-8)
Supplement: Supplementary file 4 — Description of Additional Supplementary Files [file 41467_2020_16802_MOESM4_ESM.pdf]

Description of Additional Supplementary Files

File Name: Supplementary Data 1
Description: List of the 1686 patients analyzed by whole-genome sequencing (WGS), and classification of their APOBEC expression levels and mutation signatures

File Name: Supplementary Data 2
Description: List of the 77 patients analyzed by whole-exome sequencing (WXS) and RNA-Seq, and their APOBEC3A expression levels

File Name: Supplementary Data 3
Description: List of the 50 top RNA editing hotspots identified, and their percent editing in the APOBEC3A-low and APOBEC3A-high cohorts

File Name: Supplementary Data 4
Description: List of blood cancer types of patient samples analyzed in Figure 7
